# Effect of inaccurate b‐values from imaging gradients on intravoxel incoherent motion

**DOI:** 10.1002/mrm.30579

**Published:** 2025-06-02

**Authors:** Ivan A. Rashid, Filip Szczepankiewicz, Adalsteinn Gunnlaugsson, Lars E. Olsson, Patrik Brynolfsson

**Affiliations:** ^1^ Department of Translational Medicine, Medical Radiation Physics Lund University Malmö Sweden; ^2^ Department of Medical Radiation Physics, Clinical Sciences Lund Lund University Lund Sweden; ^3^ Department of Clinical Sciences Lund, Radiation Therapy Lund University Lund Sweden; ^4^ Department of Hematology, Oncology and Radiation Physics Skåne University Hospital Lund Sweden; ^5^ Hero Imaging AB Umeå Sweden

**Keywords:** accuracy, B matrix, diffusion, imaging gradients, IVIM, reproducibility

## Abstract

**Purpose:**

Neglecting imaging gradients in b‐value calculations has been a documented issue for decades and remains unaccounted for in the current postprocessing pipelines. This omission may introduce inaccuracies that propagate into diffusion parameter estimates, such as in ADC and DTI analysis. Because intravoxel incoherent motion (IVIM) makes use of low b‐values, these inaccuracies may be of greater importance. This study examines the impact of biased b‐values on IVIM analysis in simulations and in vivo.

**Methods:**

In simulations, b‐values were calculated for two pulsed gradient spin‐echo sequence designs: one with large cross‐terms between imaging and diffusion gradients, and one with minimal cross‐terms. Biased and unbiased b‐values were calculated from sequences with 200 diffusion directions. These b‐values were used to generate IVIM signal curves for parameter estimation. Simulations were repeated with varying in‐plane resolutions (1–4 mm) and slice thicknesses (2–10 mm). Additionally, 15 prostate exams were analyzed with scanner‐provided b‐values and actual b‐values derived from the gradient waveforms of the full pulse sequence.

**Results:**

The magnitude and direction of errors in IVIM parameters depended on pulse sequence design. Errors persisted until the full contribution of imaging gradients was considered. Errors in the in vivo data were coherent with the simulations, showing errors of −0.7% in *f*, 0.8 μm^2^/ms in D*, and 0.07 μm^2^/ms in *D*.

**Conclusion:**

Ignoring imaging gradients in b‐value calculations introduces unnecessary inaccuracies, making IVIM results spurious and highly dependent on specific pulse sequence design and imaging parameters. These inaccuracies can be corrected by adjusting the b‐value calculations, without additional measurements.

## 1 INTRODUCTION

Since the early days of diffusion MRI, intravoxel incoherent motion (IVIM) modeling has faced several challenges, most prominently its poor repeatability and reproducibility.^1^, ^2^, ^3^ Despite this, it is recognized to have several promising applications throughout the body,^1^, ^3^, ^4^ prompting efforts to improve the reliability of IVIM parameter estimates.^3^ Recent improvements include optimization of b‐value distributions,^5^, ^6^, ^7^, ^8^, ^9^ development and evaluation of traditional and machine‐learned fitting algorithms,^10^, ^11^, ^12^, ^13^ advanced diffusion encoding and modeling,^8^, ^14^, ^15^ and accounting for sources of bias such as T_2_ relaxation^16^ and signal drift.^17^

Another often‐neglected bias in DWI is the effect of imaging gradients on the diffusion weighting (b‐value). Indeed, imaging gradients can both increase and decrease the desired b‐value depending on their interaction with the diffusion encoding gradients.^18^ Moreover, unlike the diffusion gradients, the imaging gradients cannot be turned off, which means that the b‐value cannot be exactly equal to zero.^2^ Despite this, it is common to assume that the diffusion weighting is exactly zero for an image “without diffusion weighting” as it is often reported in the metadata of the DWI. This oversight may be viable in the context of other DWI applications but could be important for IVIM because the model aims to extract information from signals acquired at relatively low b‐values (<200 s/mm^2^).

Because b‐values of exactly 0 s/mm^2^ are given as outputs in the metadata, it is reasonable to investigate the accuracy of b‐values reported by both clinical and research implementations of DWI pulse sequences, as they may differ from the actual diffusion weighting of the images. It is also expected that the bias differs between pulse sequence implementations because it is inherently a consequence of pulse sequence design in terms of the timing and order of magnetic field gradients.^19^ These methodological differences result in reduced single‐ and multi‐site reproducibility and need to be considered in the development of imaging biomarkers,^20^ especially in examples of applications in which quantitative IVIM parameter values are of interest.^21^

The effect of imaging gradients has been studied for ADC,^19^, ^22^, ^23^, ^24^ DTI,^25^, ^26^, ^27^, ^28^, ^29^ and more advanced DWI applications such as q‐space MRI^30^ and high angular resolution diffusion imaging.^31^ It has been consistently found that imaging resolution and pulse sequence design are important factors, and that accounting for these effects in acquisition or postprocessing improves accuracy. Several studies have shown that the errors in diffusion weighting are especially severe when imaging and diffusion encoding gradients both contribute to the dephasing vector simultaneously, leading to so‐called cross‐term interactions.^25^, ^27^, ^29^, ^30^, ^32^ These interactions can increase or decrease the diffusion weighting, making the pulse sequence design important.^18^, ^19^ Furthermore, the gradients depend on the imaging parameters, such as image resolution,^33^ which affect the magnitude of the errors.^18^

Although well studied for other DWI applications, the literature is scarce for IVIM. Yuan et al.^34^ simulated pulse sequences of high‐resolution acquisitions and found that the pseudo‐diffusion coefficient D* would benefit the most from using actual b‐values in IVIM analysis, whereas the effects on the perfusion fraction *f* and diffusion coefficient *D* were small. Szczepankiewicz and Sjölund^35^ investigated the bias imposed by so‐called background gradients, which have similar effects on the diffusion signal as imaging gradients, and found that all IVIM parameters would benefit from a correction. To our knowledge, no extensive evaluation on the influence of biased b‐values from imaging gradients has been performed on IVIM modeling of DWI data. Therefore, the aim of this work is to investigate the bias on the b‐values imposed by implementations of pulsed gradient spin‐echo (PGSE) sequences and its propagation to the IVIM parameter estimates.

## 2 THEORY

### 2.1 The actual diffusion encoding of a pulse sequence

The b‐value is given by the trace of the b‐tensor (also known as the B matrix), *b* = *tr*(**B**). Therefore, to evaluate what effect different gradient components of a pulse sequence have on the b‐value, the b‐tensor needs to be decomposed into the contributions from imaging and diffusion gradients. To do this, we consider the effective gradient as a function of time g(t) (including the sign change after the refocusing pulse) between time of excitation and the TE as^26^, ^36^

(1) $$g\left(t\right)=g_{\mathrm{img}}\left(t\right)+g_{\text{diff}}\left(t\right),$$

where each gradient is a time‐varying vector along three spatial axes $\left[g_{x}\left(t\right)\;g_{y}\left(t\right)\;g_{z}\left(t\right)\right]$. From this, it follows that the spin dephasing vector q(t)=γ∫g(t)dt is defined as the sum of the dephasing vectors imposed by the imaging and diffusion gradients,^35^ such that $q\left(t\right)=q_{\mathrm{img}}\left(t\right)+q_{\text{diff}}\left(t\right)$.

The b‐tensor of a full pulse sequence is given by the dephasing vector as^26^, ^37^

(2) $$B=\int_{0}^{\mathrm{TE}}q\left(t\right)⊗q\left(t\right)dt,$$

where ⊗ is the outer product, and the integration bounds from the time of excitation (t = 0) to the TE. Because q(t) is a sum of two components, the distributive property of the outer product gives the b‐tensor as a sum of three components^35^

(3) $$B=B_{\mathrm{img}}+B_{\text{diff}}+B_{\mathrm{ct}},$$

where **B**_img_ is the b‐tensor from **g**_img_, **B**_diff_ the b‐tensor from **g**_diff_, and **B**_ct_ the resulting tensor from the cross‐term interactions between **g**_img_ and **g**_diff_. Equation (2) gives each component as^35^

(4) $$B_{\mathrm{img}}=\int_{0}^{\mathrm{TE}}q_{\mathrm{img}}\left(t\right)⊗q_{\mathrm{img}}\left(t\right)dt,$$

(5) $$B_{\text{diff}}=\int_{0}^{\mathrm{TE}}q_{\text{diff}}\left(t\right)⊗q_{\text{diff}}\left(t\right)dt,$$

(6) $$\begin{array}{c} B_{\mathrm{ct}}=\int_{0}^{\mathrm{TE}}q_{\mathrm{img}}\left(t\right)⊗q_{\text{diff}}\left(t\right)dt+\int_{0}^{\mathrm{TE}}q_{\text{diff}}\left(t\right)⊗q_{\mathrm{img}}\left(t\right)dt. \end{array}$$

Given a set of imaging parameters, an imaging pulse sequence can be generated. These could be invariant during the image acquisition, yielding a constant **B**_img_. However, **B**_diff_ is often changed from shot to shot such that the amplitude, timing, and direction of **g**_diff_ are modulated. This leads to changes in **B**_diff_ and **B**_ct_, which need to be accounted for.^18^

### 2.2 Cross‐term interactions between imaging and diffusion gradients

According to Equation (6), imaging and diffusion gradients interact with each other when their dephasing vectors $q_{\mathrm{img}}\left(t\right)$ and $q_{\text{diff}}\left(t\right)$ are not orthogonal. A design condition that helps to minimize these interactions as well as the trace of **B**_img_ is to keep $q_{\mathrm{img}}$ at zero for as long as possible for the duration of the pulse sequence. This is achieved by immediate prewinding and rewinding of imaging gradients.^18^

Prewinders and rewinders are gradients that revert the additional dephasing caused by imaging gradients in order to return to the center of the k‐space.^33^ In typical PGSE implementations, rewinders are used for the slice‐selective excitation gradient, whereas prewinders are used for the crushers and the EPI readout train.^33^

The position of the prewinder and rewinder gradients are important in terms of the diffusion weighting. Because diffusion weighting accumulates for as long as q(t) is non‐zero, not rewinding immediately after a gradient block, or prewinding immediately before one, will result in larger b‐value contributions from the imaging gradients and cross‐terms.^18^, ^19^ Such effects can occur in sequences that were designed to optimize TE.^33^ For example, the slice‐selective rewinder can be merged with the crusher prewinder, as opposed to having two gradient blocks performing these tasks as close as possible to the imaging gradients in question. In such a case, some compromise would have been made on either the position of the rewinder or prewinder, leading to prolonged non‐zero $q_{\mathrm{img}}$ and a larger b‐value contribution.

## 3 METHODS

### 3.1 Simulations

To evaluate the range of errors on the reported b‐values and resulting IVIM parameter estimates imposed by neglecting imaging gradients, three scenarios were conceptualized using Equation (3) and are summarized in Table 1. These were used for calculating b‐values, including different contributions from the gradients and their interactions.

**TABLE 1:** The three scenarios for b‐value calculations used in this work. They were chosen to isolate and study the effect of considering only a subset of contributions to the b‐tensor as described by Equation (3).

Label	b‐tensor definition	Description
Nominal	**B**_diff_	Only the diffusion gradients are assumed to contribute to the diffusion weighting.
Accounting for imaging	**B**_diff_ + **B**_img_	Diffusion weighting is assumed to be caused by the diffusion gradients and the imaging gradients with no interactions between them.
Accounting for imaging and cross‐terms	**B**_diff_ + **B**_img_ + **B**_ct_	Diffusion weighting caused by the diffusion gradients, imaging gradients, and cross‐term interactions. The result of this calculation would be the actual b‐value given by the pulse sequence.

*Note*: The definitions for each term are given by Equations ((4), (5), (6))–((4), (5), (6))

The simulations were limited to slice‐selective, crusher, and pre‐/rewinder gradients because these are the gradients that cause meaningful cross‐term interactions with the diffusion gradients that are propagated to the resulting b‐value.^19^, ^25^, ^34^, ^36^, ^38^ Furthermore, we restricted ourselves to PGSE sequences because these are the most widely used in DWI.^39^ Two examples of pulse sequence implementations were chosen for evaluation:

- A sequence with large cross‐terms. The excitation rewinder was merged into the crusher prewinder, combined with crusher gradients that were always applied regardless of the diffusion gradients.
- A sequence with minimal cross‐terms, for which rewinding was performed immediately after excitation, and crushers were only applied when the diffusion gradients alone did not provide enough dephasing for crushing of spurious echoes.

Both sequence designs are commercially available by major vendors, making them relevant options for evaluation.

#### 3.1.1 Simulation of imaging gradients

The gradient waveforms of both pulse sequences were generated using an in‐house Python script. For guidance, two commercial DWI sequences were studied (not shown) to make our evaluation applicable to current real‐world scenarios.

The gradient amplitude for slice selection during the excitation and refocusing RF‐pulses was defined as^33^

(7) $$G_{z}=\frac{2\pi }{\gamma }\frac{∆f}{∆z},$$

where ∆f is the transmit bandwidth and ∆z the slice thickness. A transmit bandwidth of 600 Hz was used, with a rectangular shape for the gradient. The durations of the excitation and refocusing slice‐selective pulses were 6 and 7 ms, respectively.

Triangular shapes were used for the crushers and pre‐/rewinders, with a target de‐/rephasing ∆ϕ defined as^33^

(8) ∆ϕ=γ∆r∫G(t)dt,

where ∆r is the voxel size in the gradient direction, and G(t) the gradient amplitude as a function of time for the single gradient lobe in question. For crushing, the ∆ϕ was chosen as 6π, whereas ∆ϕ for the pre‐/rewinders were governed by their respective imaging gradients according to the condition $∆\phi_{\mathrm{img}}+∆\phi_{\mathrm{pre}/\mathrm{re}}=0$.

**FIGURE 1:**
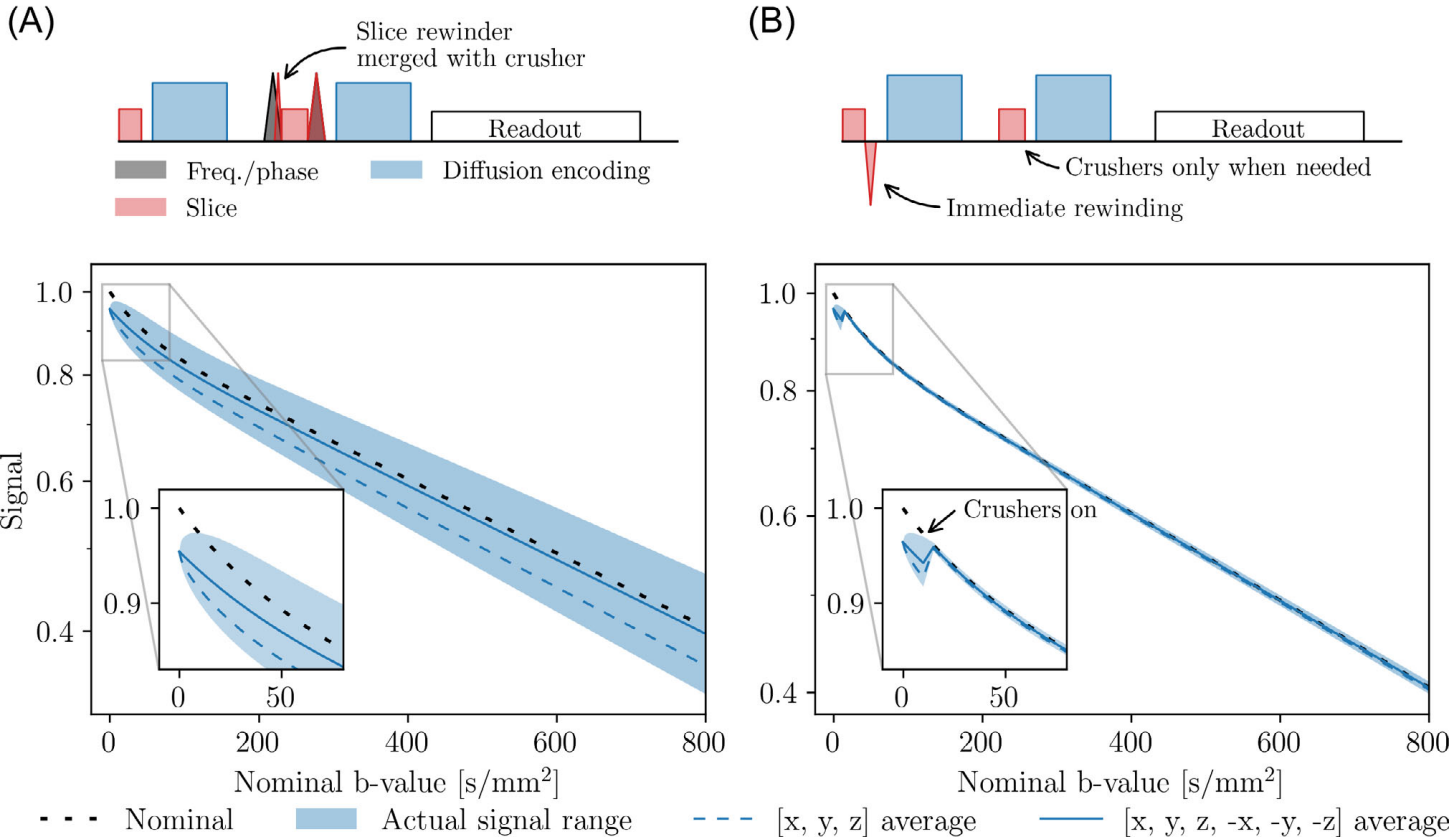
Signal deviations of two different pulse sequence designs: a sequence with large cross‐terms (A), and a sequence with minimal cross‐terms (B). The [*x*, *y*, *z*] average shows the directionally averaged signal curve for the most common IVIM analysis, whereas [*x*, *y*, *z*, −*x*, −*y*, −*z*] average shows the effect of experimental cross‐term correction by acquiring antipodal directions. The sequence with large cross‐terms is designed without considering the cross‐term interactions between the imaging and diffusion gradients, leading to a larger bias in the b‐value. When the acquired signals are mapped to the nominal b‐values, a bias is engraved into the data, manifesting as a signal deviation. Because the cross‐term interactions depend on the diffusion encoding direction, a range of actual signal curves will be obtained. Furthermore, the configuration of the imaging gradients affects the magnitude of the bias, hence a difference in signal ranges between pulse sequence designs. Signals were simulated for 200 diffusion directions well distributed over a sphere, with a 1 mm isotropic imaging resolution. IVIM, intravoxel incoherent motion.

Figure 1 shows the resulting pulse sequence designs for an isotropic resolution of 1 mm. No EPI readout blocks were simulated because their contributions to the b‐value were assumed to be negligible due to the high‐frequency gradient oscillations.^26^ The gradient waveforms were defined with a 12 μs temporal resolution.

#### 3.1.2 Simulation of diffusion gradients

For diffusion encoding, 200 well‐distributed directions over a sphere^40^ were simulated as rectangular blocks with constant diffusion gradient duration δ = 20 ms and diffusion gradient separation Δ = 40 ms. Gradient amplitudes were set to achieve 34 nominal b‐values ranging between 0 and 800 s/mm^2^, with tighter sampling for lower b‐values (0, 1, 2, 3, 4, 5, 6, 7, 8, 9, 10, 15, 20, 25, 30, 35, 40, 45, 50, 60, 70, 80, 90, 100, 125, 150, 175, 200, 300, 400, 500, 600, 700, 800 s/mm^2^).

In addition to the 200 well‐distributed directions, two direction sets were simulated to study directionally averaged signals: a standard three‐directional set (*x*, *y*, *z*) and a six‐directional set with experimental cross‐term correction using antipodal directions^25^ (*x*, *y*, *z*, −*x*, −*y*, −*z*).

#### 3.1.3 Evaluation of b‐value errors on IVIM parameter estimates

Simulations were performed for a range of slice thicknesses and in‐plane resolutions to evaluate the effect of each of the imaging parameters on the b‐value separately. For varying slice thickness, the in‐plane resolution was kept constant at 2 × 2 mm^2^, whereas the slice thickness was varied from 2 mm to 10 mm (step size 1 mm). For varying in‐plane resolution, the slice thickness was kept constant at 4 mm, whereas the in‐plane resolution was varied from 1 × 1 mm^2^ to 4 × 4 mm^2^ (step size 0.25 × 0.25 mm^2^). Additionally, simulations were performed with isotropic resolutions ranging from 1 mm to 4 mm (step size 0.25 mm).

The scenarios described in Table 1, along with Equations ((4), (5), (6))–((4), (5), (6)), were used to calculate b‐values under different assumptions. The b‐values in the set that accounted for both imaging and cross‐terms were regarded as the actual b‐values, hence used as the reference in the evaluation.

Signals were calculated using the bi‐exponential IVIM model

(9) $$S\left(b\right)=S_{0}\left[fe^{-bD^{*}}+\left(1-f\right)e^{-\mathrm{bD}}\right],$$

with the reference b‐values, and ground truth parameter values *f* = 10%, D* = 20 μm^2^/ms, *D* = 1 μm^2^/ms. These values were chosen as representative for the prostate^41^ to match the in vivo measurements.

For a more general evaluation of errors, signals were calculated with three different parameter sets, each with one variable adjusted in 50 steps, whereas the others remained constant. The *f* was varied from 1% to 30%, with D* held constant at 20 μm^2^/ms and *D* at 1 μm^2^/ms. The D* varied from 5 μm^2^/ms to 50 μm^2^/ms, with *f* held constant at 10% and *D* at 1 μm^2^/ms. The *D* varied from 0.5 μm^2^/ms to 3 μm^2^/ms, with *f* at 10% and D* at 20 μm^2^/ms.

To estimate the bias in IVIM parameter estimates, the signals were mapped to the assumed b‐values of the scenarios in Table 1, and the IVIM model of Equation (9) was fitted using a single‐step nonlinear least‐squares algorithm with the ground truth values as the initial guess.

### 3.2 Application on in vivo measurements

#### 3.2.1 In vivo measurements

Five patients with biopsy‐confirmed prostate cancer were scanned longitudinally on three separate occasions: a baseline scan before treatment, and two during treatment with a gonadotropin‐releasing hormone agonist and radiotherapy. The patients were classified as very high risk according to at least two of the following criteria: T3, Gleason grade 8, prostate‐specific antigen 20–49 μg/L or Gleason score 9–10 and/or prostate‐specific antigen ≥40 μg/L. The study was approved by The Swedish Ethical Review Authority (no. 2023–00088).

In vivo measurements were performed on a 3 T Signa Architect (GE Healthcare, Milwaukee, WI), software version MR30.0_R01. A commercial DWI‐SE‐EPI sequence with reduced FOV, spatially selective excitation, and chemical shift selective fat saturation was used. Axial FOV were used with matrix size 160 × 80, in‐plane resolution 1.5 × 1.5 mm^2^, slice thickness 3 mm, with TE 69.2 ms and TR 5000 ms. The diffusion experiment was performed with conventional Stejskal‐Tanner^42^ encoding with nominal b‐values 50, 200, 800 s/mm^2^ in the *x*, *y*, and *z* directions, each with five repetitions, as well as a nominal b0 with 15 repetitions. The diffusion gradient duration was δ = 22 ms and gradient separation Δ = 31 ms.

#### 3.2.2 Determining the actual b‐values

The gradient amplitudes of the full pulse sequence, that is, the sum of both imaging and diffusion gradients, were recorded for each excitation during phantom scans and saved as discretized **g**(*t*) arrays using software provided by the vendor. This was done once for each subject using identical scanning parameters as the in vivo exams (including non‐imaging parameters such as body weight) because slight variations in the imaging gradients were observed between subjects. This process retrospectively reproduced the pulse sequences that were used during the in vivo exams on an individual basis.

The actual b‐values were calculated with the effective **g**(*t*) arrays (accounting for time of excitation and amplitude inversion after the refocusing RF pulse) according to Equation (2), including all gradients between the excitation time and the TE.

#### 3.2.3 Image analysis

Image analysis was performed using Hero version 2024.2.0 (Hero Imaging, Umeå, Sweden) and Python 3.11.5. The DWI volumes were reconstructed with the vendor‐specific denoising reconstruction AIR Recon DL (GE Healthcare, Milwaukee, WI).^43^ Rigid registration using Elastix version 4.9 was applied for intervolume motion correction prior to arithmetic averaging of signals across diffusion encoding directions.

Contrary to the simulations, due to the noise in the in vivo data, IVIM parameters were estimated using a segmented fitting approach^2^ implemented in Hero (Hero Imaging), with the b‐value threshold at 200 s/mm^2^. Two fits were performed: one with the signals mapped to the nominal b‐values, and one with the signals mapped to the actual b‐values.

The fitting algorithm was implemented as follows. First, an estimate of *D* was produced with a linear fit on the logarithm of the signal to b‐values above the threshold of 200 s/mm^2^. The signal was extrapolated to low b‐values and subtracted from the total. Second, a nonlinear estimation was performed on the subtracted signal to get initial estimates D* and *f*. Third, *D* was fixed in a final estimation of *S*_*0*_, *f*, and D*, performed using a nonlinear least‐squares algorithm on the original signal, with the previous estimates D* and *f* estimates as initial guesses.

For a quantitative evaluation, 4000 voxels from whole‐prostate region of interest were randomly sampled from the total dataset of 15 exams (46,713 voxels in the complete dataset) for a comparison between the parameter estimates from the fits with the nominal and actual b‐values.

## 4 RESULTS

### 4.1 Simulations

Imaging gradients caused the signal to deviate from its assumed nominal shape (Figure 1). The simulations of 200 well‐distributed diffusion directions generated a signal range encompassing signal curves with both negative and positive bias. In the directional averages, the signal curves showed a constant deviation reflecting the average bias of the used diffusion directions. The size of the signal range and the deviation of the directionally averaged signal curves depended on pulse sequence design.

**FIGURE 2:**
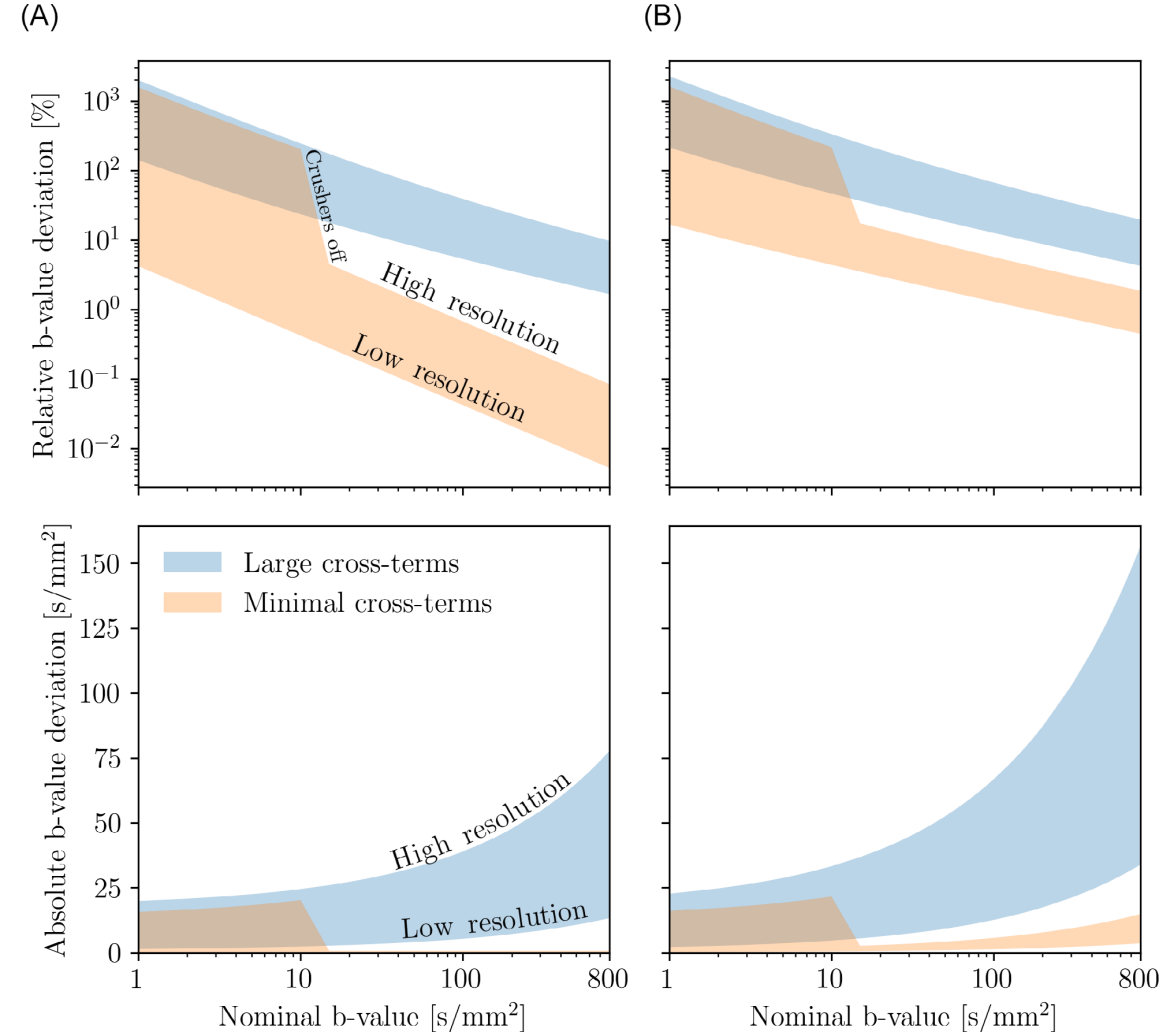
Relative (upper row) and absolute (lower row) b‐value deviations as a function of b‐value, shown for both the sequence with large cross‐terms, and the sequence with minimal cross‐terms. The possible errors are shown as ranges between the highest resolution (1 mm isotropic) and lowest resolution (4 mm isotropic). (A) Deviations with the diffusion gradients on the *x*‐ or *y*‐axis. (B) Deviations with the diffusion gradients on the *z*‐axis.

The relative deviations found in the b‐values depended on the b‐values themselves, where the errors decreased with increasing b‐value (Figure 2). Errors could range between 100 and 1000% in the ultralow b‐value range (<10 s/mm^2^) depending on image resolution (Figure 2). Furthermore, there was a large dependence on pulse sequence design. In the sequence with minimal cross‐terms, once the diffusion gradients were large enough for the crushers to be turned off (b‐value >10 s/mm^2^), the worst‐case (high‐resolution) relative b‐value error was reduced from over 200% to lower than 10%. Additionally, Figure 2 shows that in a three‐directional [*x*, *y*, *z*] acquisition, the largest b‐value deviations will occur when the diffusion gradients are on the *z*‐axis due to the additional interactions with the slice‐selective imaging gradients.

**FIGURE 3:**
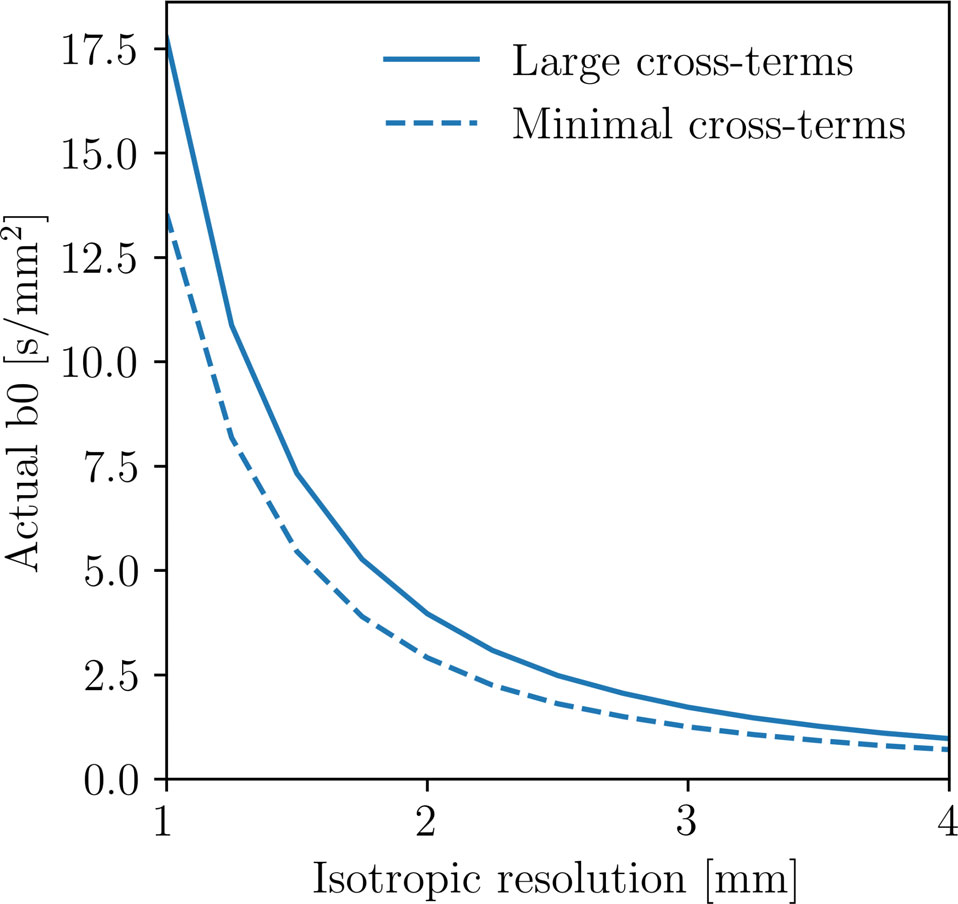
The actual b‐value of an “unweighted” diffusion image (b0) as a function of image resolution, simulated for both a sequence with minimal and large cross‐terms. This figure shows the minimal achievable b‐value for these pulse sequences at the given image resolutions.

Depending on image resolution, Figure 3 shows that ultralow b‐values can be impossible to achieve due to the actual b‐value of a pulse sequence without diffusion gradients. For a 1 mm isotropic resolution, the minimum b‐value was 13.5 s/mm^2^ with minimal cross‐terms. For lower resolutions such as 3 mm isotropic, the minimum b‐value was 1.25 s/mm^2^.

**FIGURE 4:**
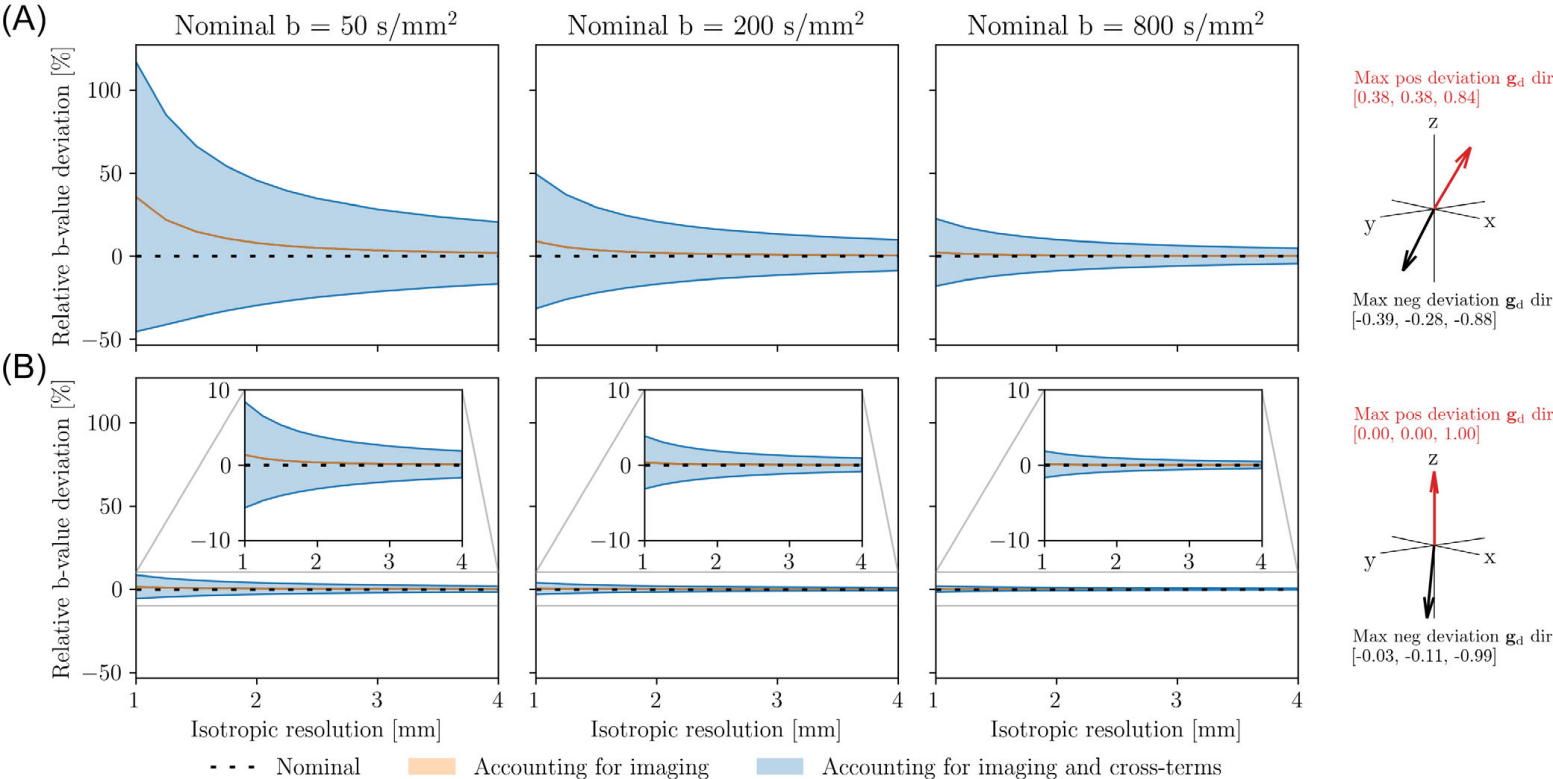
The relative deviation from the nominal b‐value for a subset of the 34 simulated b‐values as a function of isotropic resolution for a sequence with large cross‐terms (A) and sequence with minimal cross‐terms (B). The b‐values were chosen as representative for the low (50 s/mm^2^), intermediate (200 s/mm^2^), and high (800 s/mm^2^) b‐value range in the context of IVIM. The error ranges are shown for 200 well‐distributed diffusion directions over a sphere. The b‐values were calculated according to the scenarios in Table 1. The diffusion directions for the worst‐case positive and negative errors are shown as vectors.

Figure 4 shows how the b‐value deviations evolve by accounting for imaging and cross‐terms. When we accounted for imaging gradients by adding the **B**_img_ term, the b‐value increased depending on the image resolution and was independent of the diffusion direction. When the cross‐terms were included with the **B**_ct_ term, thus performing a full calculation, both positive and negative errors dependent on the diffusion direction were revealed. For the lowest resolution (4 mm isotropic), b‐value 50 s/mm^2^ varied by up to ±25% depending on diffusion direction. This shows that cross‐terms are a key source of b‐value error.

**FIGURE 5:**
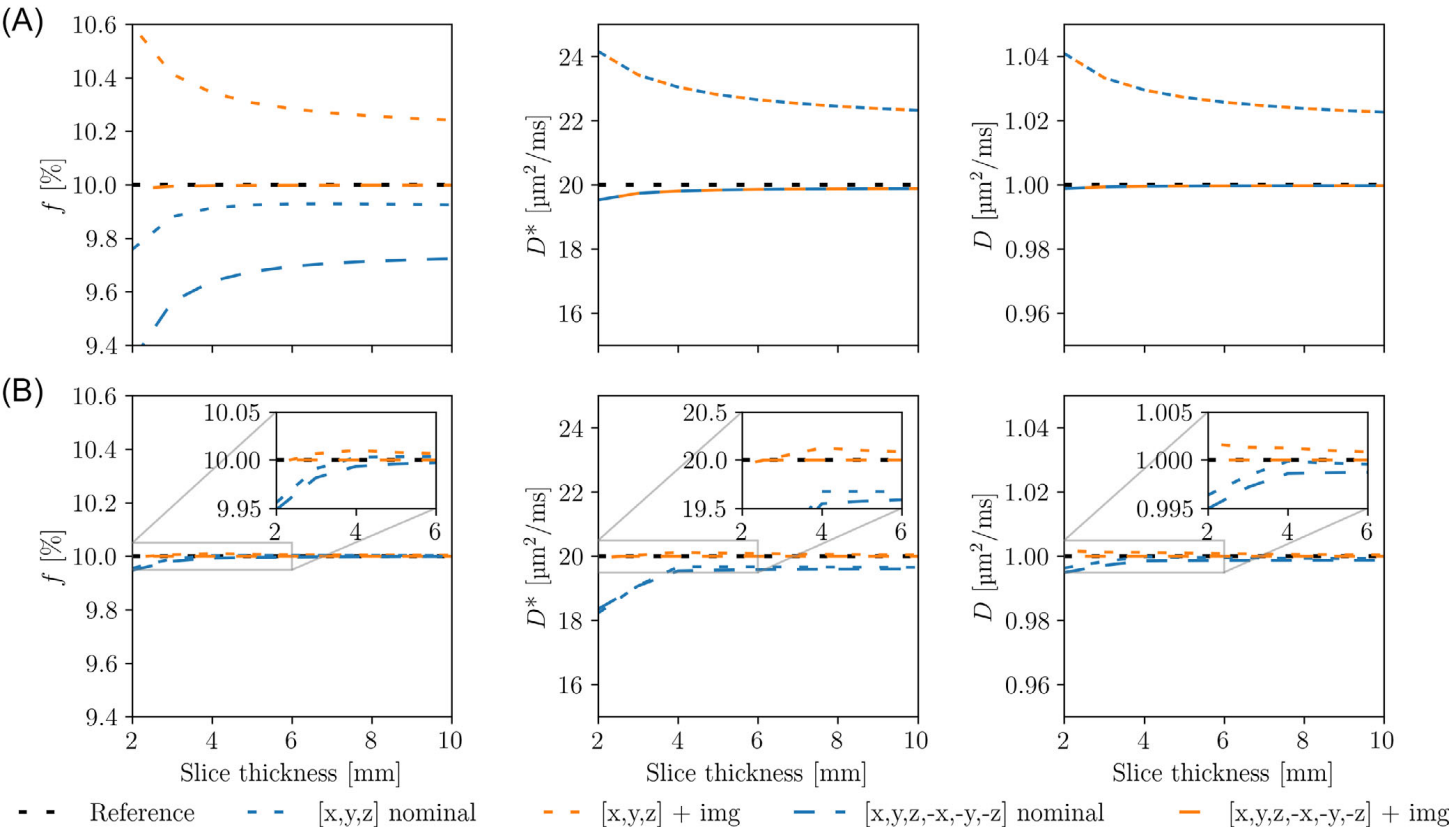
IVIM errors versus slice thickness. Simulated IVIM parameter estimates produced from a pulse sequence with large cross‐terms (A) and minimal cross‐terms (B) as a function of slice thickness using different sets of diffusion directions and b‐value calculation methods. The in‐plane resolution was fixed to 2 × 2 mm^2^. The lines show the fits to geometrically averaged signals for two different diffusion direction sets, with (orange) and without (blue) experimental cross‐term correction. The reference line shows fits performed with the actual b‐values including both imaging and cross‐terms, which were assumed as the ground truth. The signals were calculated using a large set of 34 b‐values between 0 and 800 s/mm^2^ (nominal) with tighter sampling in the lower b‐value range.

**FIGURE 6:**
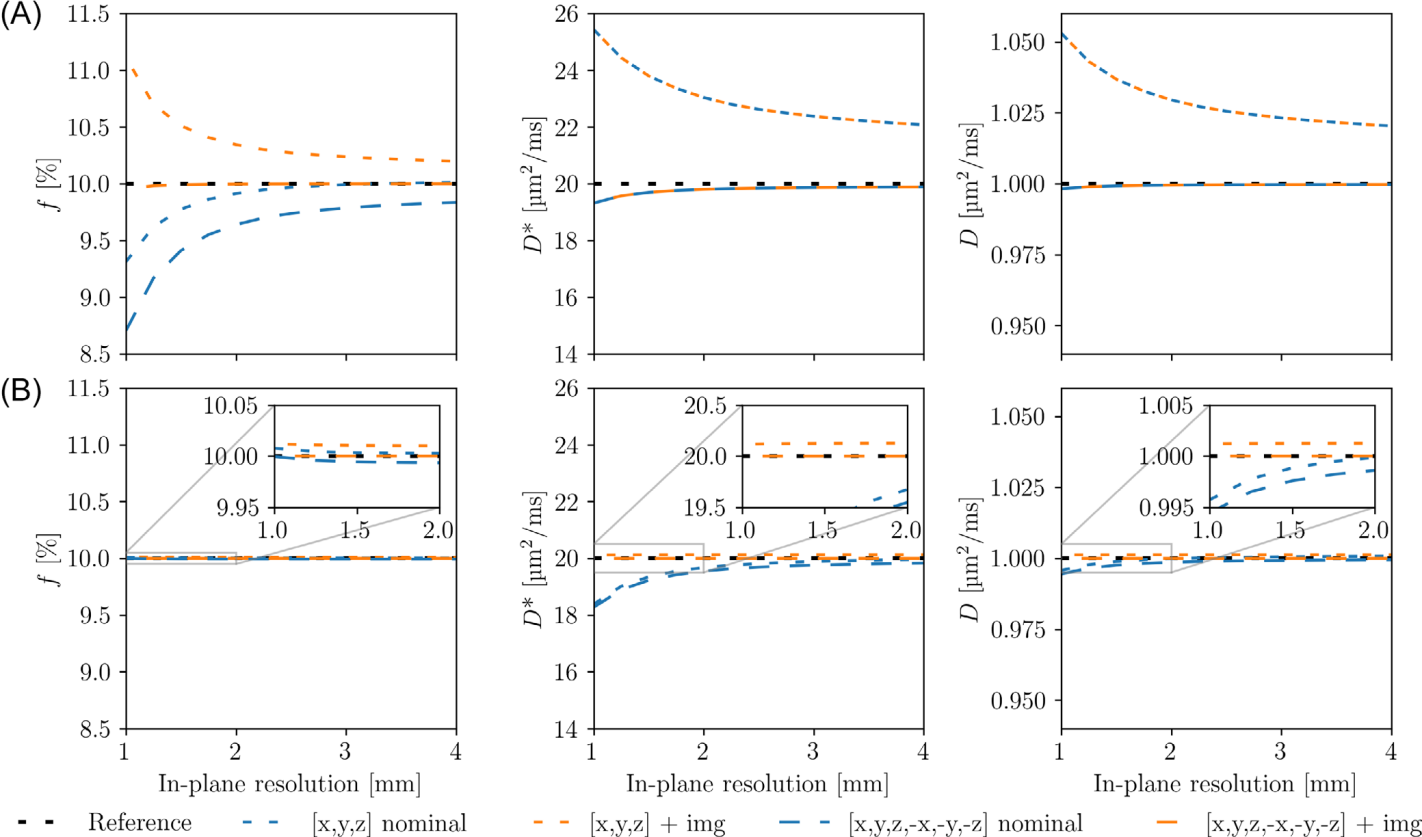
IVIM errors versus in‐plane resolution. Simulated IVIM parameter estimates produced from a pulse sequence with large cross‐terms (A) and minimal cross‐terms (B) as a function of in‐plane resolution using different sets of diffusion directions and b‐value calculation methods. The slice thickness was fixed 4 mm. The lines show the fits to geometrically averaged signals for two different diffusion direction sets, with (orange) and without (blue) experimental cross‐term correction. The reference line shows fits performed with the actual b‐values including both imaging and cross‐terms, which was assumed as the ground truth. The signals were calculated using a large set of 34 b‐values between 0 and 800 s/mm^2^ (nominal), with tighter sampling in the lower b‐value range.

**FIGURE 7:**
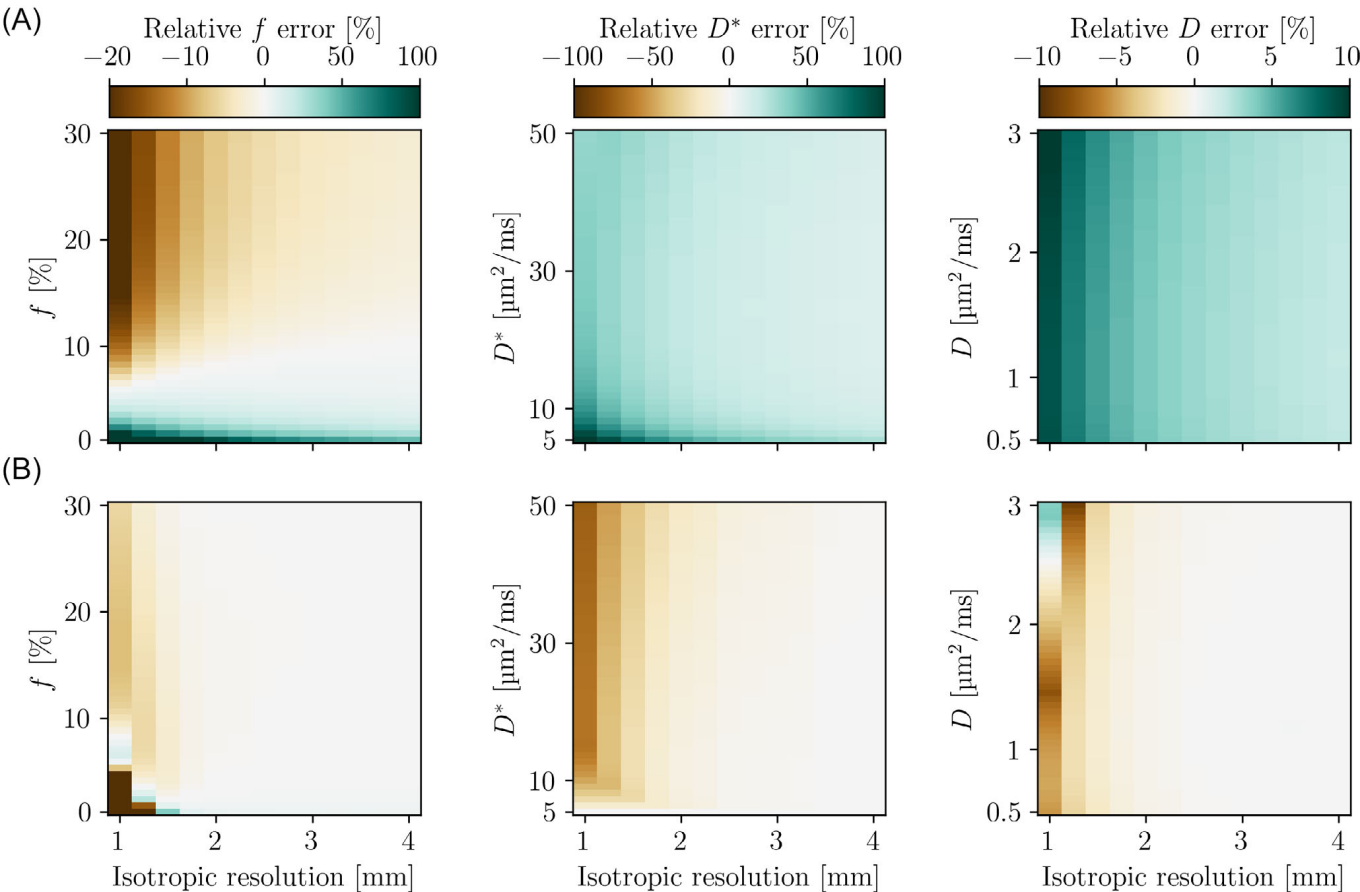
The relative errors of IVIM parameter estimates for different parameter sets for simulated sequences with large cross‐terms (A) and minimal cross‐terms (B). Signals were calculated with one varying parameter at a time, keeping the other two constant. The default values were *f* = 10%, D* = 20 μm^2^/ms, and *D* = 1 μm^2^/ms. The extreme values seen for the *f* estimates in the sequence with minimal cross‐terms (bottom left) were −100%.

IVIM analysis with nominal b‐values resulted in both positive and negative errors in the parameter estimates. The magnitude of these errors depended on pulse sequence design, diffusion directions, slice thickness (Figure 5), in‐plane resolution (Figure 6), and parameter value (Figure 7). For both pulse sequences, the bias in the parameter estimates were eliminated when the imaging component **B**_img_ was included together with the experimental cross‐term correction using antipodal diffusion‐encoding directions (Figures 5 and 6).

When the imaging and the cross‐terms were not considered, IVIM parameter estimates were biased by varying sign and magnitude, which depended on the pulse sequence design (Figure 7). For the sequence with large cross‐terms, D* and *D* always had a non‐zero relative error of at least 10% and 2%, respectively. However, with a sequence designed for minimal cross‐terms, negligible errors were attainable for resolutions lower than 2 mm isotropic. For *f*, the errors were both positive and negative, depending on the parameter value and pulse sequence.

### 4.2 Application on in vivo measurements

The relative b‐value error was approximately 6% across all b‐values (Table 2). Considering that the pulse sequence resembled the simulated sequence with large cross‐terms, these experimental errors are lower than the simulated errors demonstrated in Figures 2, 3, 4. In addition to a common larger error, a smaller variation in the first decimal was found between the subjects. This variation was connected to a slight change in the amplitude of the slice‐selective gradients, showing that b‐values may still uncontrollably differ between subjects despite using the same imaging protocol.

**TABLE 2:** The actual b‐values for each patient. The patient‐to‐patient variation is in the order of the first decimal with a positive trend between patient weight and b‐value.

Weight (kg)	Actual b0 (s/mm^2^)	Actual b50 (s/mm^2^)	Actual b200 (s/mm^2^)	Actual b800 (s/mm^2^)
73	0.44	53.33	212.02	845.77
88	0.46	53.41	212.13	845.80
89	0.47	53.41	212.12	845.82
100	0.48	53.46	212.19	845.85
117	0.49	53.23	212.24	845.87

**FIGURE 8:**
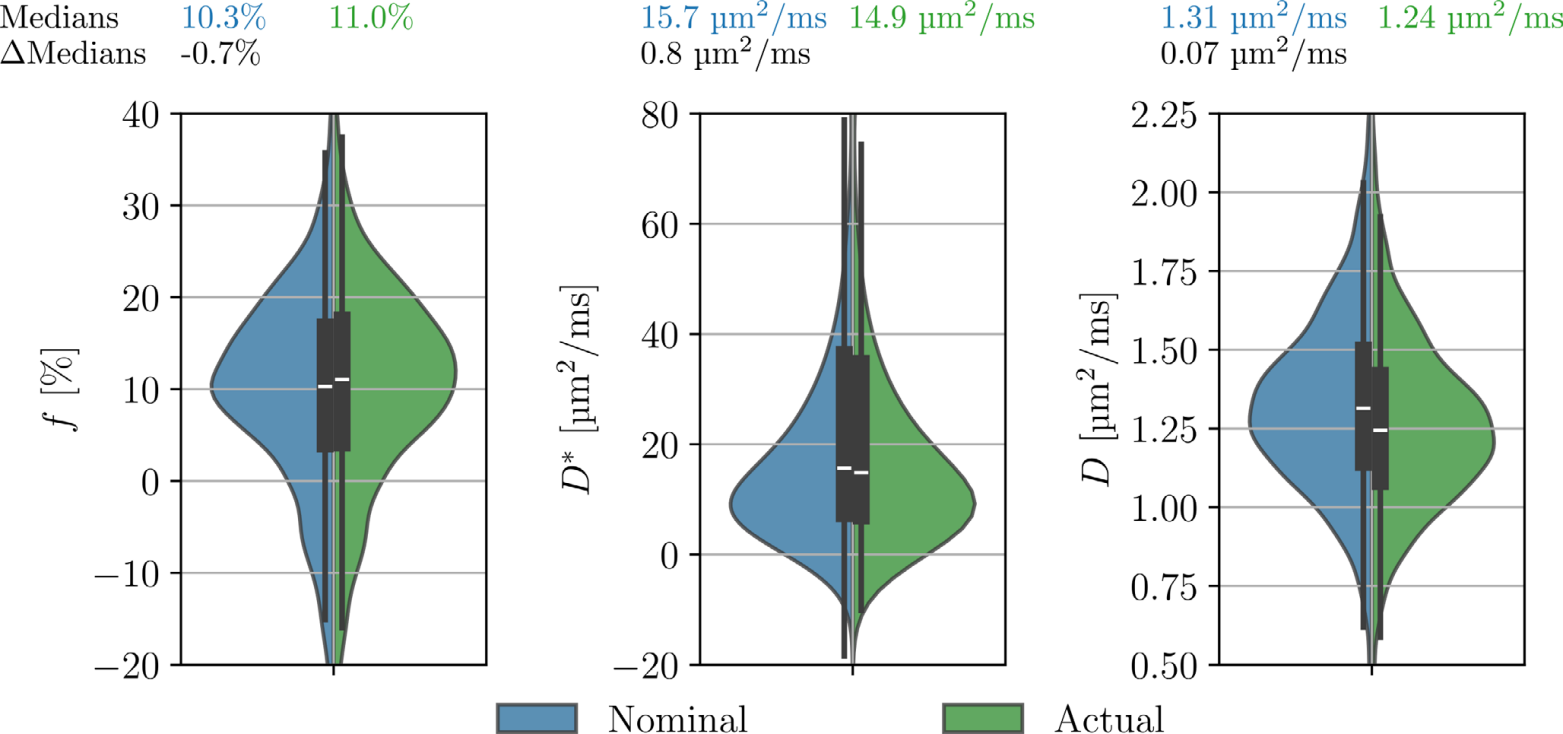
IVIM parameter distributions of 4000 voxels randomly sampled from 15 different prostate exams (total 46,713 voxels). Both the direction and the order of magnitude of the ΔMedians are consistent with the simulations of the sequence with large cross‐terms in Figures 5, 6, 7, in which the use of nominal b‐values underestimated *f* and overestimated D* and *D*. The distributions include both noise and heterogeneity as voxels were sampled from the entire prostate.

In the prostate dataset, quantitative differences were found for all three IVIM parameters when estimated using both nominal and actual b‐values (Figure 8). The differences between the medians of the distributions were −0.7% for *f*, 0.8 μm^2^/ms for D*, and 0.07 μm^2^/ms for *D*. Both the sign and magnitude of these *f* and *D* errors agree with the nominal [*x*, *y*, *z*] curves in Figures 5 and 6 for the sequence with large cross‐terms, which was the scenario most applicable to the in vivo dataset.

## 5 DISCUSSION

The use of accurate b‐values has been recommended for IVIM analysis in the past.^22^ However, to our knowledge, it has only been applied in a single contemporary IVIM study.^44^ Negligence of this source of bias in IVIM analysis could be attributed to the scarce literature on what effect it has on IVIM parameter estimates. The issue is further exacerbated by inaccessibility and lack of transparency because the actual b‐values are typically not provided in the metadata of the images from clinical MR scanners. Therefore, we sought to evaluate these errors in different scenarios, including a demonstration in vivo, to answer whether the b‐value inaccuracies need to be considered in IVIM analysis.

We have studied the effect of PGSE imaging gradients on the b‐value. We simulated imaging scenarios with three relevant variables: in‐plane resolution, slice thickness, and pulse sequence design with respect to cross‐term interactions between imaging and diffusion gradients. The errors were evaluated from the b‐value to the final IVIM parameter estimates. We have shown that all three of these experimental variables contribute to errors, prompting the need for data harmonization by accounting for all the gradients in the calculation of the b‐value. Furthermore, we demonstrated the effect of using the true b‐values in IVIM analysis in vivo on prostate cancer in a clinical setting, which largely agreed with the results of the simulations. Our results highlight the importance of using actual b‐values for IVIM analysis, which would be greatly simplified if vendors made them accessible to users of DWI pulse sequences.

### 5.1 Simulations

From the simulations, we learned that pulse sequence design plays an important role in the accuracy of the b‐value. This corroborates previous works in which pulse sequence design has been demonstrated to affect the b‐value and subsequent DWI analysis if only diffusion gradients are considered.^19^, ^29^ Because manufacturers of MR‐scanners and researchers design their pulse sequences differently, this experimental variability alone motivates the need for accurate recording of b‐values and b‐tensors.^20^ By including all the gradients from spin excitation to TE in the calculations, the differences in pulse sequence design can be accounted for.

The deviation of the directionally averaged signals can become negligibly small if a pulse sequence with minimal cross‐terms is used. This emphasizes the importance of pulse sequence design, and is indeed in line with previous studies in which cross‐terms have been highlighted as a major source of error.^19^, ^25^, ^34^, ^36^, ^38^ However, because the design is often hidden from the user, it becomes increasingly important to transparently communicate the actual b‐value; the results may otherwise be erroneous.

The only scenario in our simulations in which data was fully harmonized, and negligible errors were seen due to pulse sequence design or image resolution, was when both imaging gradients and cross‐terms were included in the analysis. We showed that by performing experimental cross‐term correction using antipodal diffusion directions together with the simple additive **B**_img_ contribution, we achieved the same results as a full calculation including all components. This implies that there is no need for experimental cross‐term correction^25^ to eliminate the bias, and that data can be harmonized by simply performing a complete b‐value calculation with Equation (2) using the information accessible in the pulse sequence program, or retrospectively if one can reproduce the exact same pulse sequence for a given dataset.

The errors in the perfusion‐related parameters *f* and D* coincide with larger relative deviations in the lower b‐values. This is likely due to **q**_diff_ approaching the size of the constant **q**_img_, making **B**_diff_ less dominant in Equation (6), with increased importance placed on **B**_img_ and **B**_ct_. Therefore, model parameters that rely on information in the low b‐value range become more affected by the usage of nominal b‐values than model parameters relying on information in the higher b‐value range, as seen by the larger relative errors for *f* and D* compared to *D* in Figure 1, 7.

A limitation of the simulations was that only two PGSE sequence designs were evaluated. Because gradients can be arranged in numerous different ways, we decided to limit this work to realistic best‐ and worst‐case scenarios. For example, an in‐between configuration could be one in which the readout prewinder is merged with the crushers rather than being performed immediately before readout. However, we ignored the EPI‐readout portion of the pulse sequence because it has previously shown negligible contributions to the b‐value^26^ due to its high frequency oscillations leading to small **q**‐values. It is expected that the most substantial b‐value contribution from EPI readout gradients would be the phase encoding gradient between the phase prewinder to the TE.^26^

A future prospect beyond the scope of this paper is to investigate the effect on advanced diffusion encoding and modeling. Current research into velocity‐compensated IVIM modeling utilizes bipolar velocity‐compensated diffusion encoding gradients,^14^, ^15^ which are less prone to cross‐terms with imaging gradients.^29^, ^45^ Indeed, these are additionally exposed to errors in their velocity encoding from the imaging gradients, which remains to be quantified.

### 5.2 Application on in vivo measurements

The first finding of the in vivo measurements was the fact that the actual b‐values we obtained using Equation (2) with the inclusion of imaging gradients were not equal to the reported b‐values in the image metadata. This confirms that there may indeed be a bias in the data from clinical pulse sequences.

Notably, the errors in the b‐values (Table 2) were found to be smaller than what was implied by the simulations (Figures 2, 3, 4). This can most likely be explained by the pulse sequence design. In the pulse sequence used for the in vivo measurements, crushers were only applied on the slice selection axis if the slice thickness was greater than or equal to 3 mm. This was indeed the case in our measurements, hence the smaller cross‐terms and smaller errors. Besides this difference, the pulse sequence used in the measurements resembled the simulated sequence with large cross‐terms in Figure 1. Another example of b‐value bias found in experimental data was presented by Schneider et al.,^44^ with negative errors ranging from 6% to 15% in the low b‐value range. This demonstrates that the errors can be of varying sign and magnitude between studies due to different software and hardware, prompting the need for case‐by‐case evaluations and data harmonization.

In addition to the common error in the b‐value, there was a slight variation between subjects, likely caused by the different body weights. Because built‐in specific absorption rate calculations preceded the scans, adjustments of the RF pulse properties likely affected the imaging gradients according to Equation (7). Other factors could also contribute to subject‐to‐subject variation, such as scanning with oblique FOV. The rotation of the FOV would have been individually set for each patient, yielding unique rotations of the imaging gradients. These are examples of situations that may motivate actual b‐value calculations on a per‐subject basis; however, our in vivo data was acquired with non‐oblique axial FOV and did not show such variations.

Whereas the errors in the experimental b‐values were smaller than those seen in the simulations, the shift in the medians of the parameter estimates was similar to that implied by the simulations. This demonstrates that even small errors in the b‐values can lead to quantitative errors in the parameters. Therefore, the usage of actual b‐values does matter whenever quantitative values are of interest, especially when comparisons are made between studies with different pulse sequence implementations and imaging resolution, where the errors could be of varying sign and magnitude. However, using actual b‐values does not solve the precision problem in IVIM. The parameter distributions in Figure 8 show large overlap due to noise, two orders of magnitude larger than the inaccuracies. On a voxel‐wise basis, the use of actual b‐values may therefore have little effect. But whenever measures of central tendency become part of the analysis, inaccuracies caused by nominal b‐values should be taken into consideration.

A limitation in our in vivo evaluation is that we only performed measurements on a single MR scanner and pulse sequence implementation. However, our simulations cover several different scenarios to showcase the possible differences in such a comparison. Additionally, we believe the comparison with Schneider et al.^44^ is sufficient to show that variability exists between real measurements and should be considered in future analysis.

There are additional possibilities for further investigation into IVIM parameter estimation. Different fitting algorithms can have varying sensitivities to biased b‐values when estimating parameters. Additionally, algorithms based on deep learning could also be impacted by this error, for example, when discrepancies in bias occur between training data and inference data. These aspects of IVIM parameter estimation remain to be explored.

## 6 CONCLUSIONS

The imaging gradients and their cross‐terms with the diffusion gradients cause errors in the b‐values which, when not accounted for, result in a loss of accuracy in the IVIM parameters. The sign and magnitude of these errors depend on the pulse sequence design and the setup of imaging parameters, which can be highly heterogeneous in a multi‐site setting. Including all the imaging gradients in the calculation of the b‐value improves the accuracy, and therefore the reproducibility and comparability, of IVIM results.

## FUNDING INFORMATION

Funding support was provided by The Swedish Cancer Society (grants 21 1910S, 22 2011 Pj, 22 0592 JIA), with additional support provided by *Allmänna sjukhusets i Malmö Stiftelse för bekämpande av cancer*; *Fru Berta Kamprads stiftelse för utforskning och bekämpning av cancersjukdomar, Lund*; *Onkologiska klinikens stiftelse för bekämpande av cancer, Malmö*; and The Swedish Prostate Cancer Association.

## CONFLICTS OF INTEREST STATEMENT

Patrik Brynolfsson is a cofounder and shareholder of Hero Imaging AB and is an active developer of the Hero platform.

## Data Availability

Code and data used for the simulations, along with partial code for experimental b‐value calculations can be found on GitHub (https://github.com/IvanARashid/Rashid_MRM_2025_IVIM_bvalue_bias). Not included are the parts of the code that turns vendor‐specific files into **g**(*t*) arrays for actual b‐value calculations. The segmented fitting algorithm used for the in vivo data is part of the commercial Hero Imaging analysis software (https://heroimaging.com).
